# Neurocognitive impairment in Ugandan children with sickle cell anemia compared to sibling controls: a cross-sectional study

**DOI:** 10.3389/fstro.2024.1372949

**Published:** 2024-04-15

**Authors:** Paul Bangirana, Amelia K. Boehme, Annet Birabwa, Robert O. Opoka, Deogratias Munube, Ezekiel Mupere, Phillip Kasirye, Grace Muwanguzi, Maxencia Musiimenta, George Ru, Nancy S. Green, Richard Idro

**Affiliations:** ^1^Department of Psychiatry, Makerere University College of Health Sciences, Kampala, Uganda; ^2^Global Health Uganda, Kampala, Uganda; ^3^Department of Neurology, Columbia University Vagelos Medical Center, New York, NY, United States; ^4^Department of Mental Health and Community Psychology, Makerere University College of Humanities and Social Sciences, Kampala, Uganda; ^5^Department of Paediatrics and Child Health, Makerere University College of Health Sciences, Kampala, Uganda; ^6^Directorate of Paediatrics and Child Health, Mulago National Referral Hospital, Kampala, Uganda; ^7^Department of Pediatrics, Columbia University Vagelos Medical Center, New York, NY, United States

**Keywords:** sickle cell anemia, neurocognition, neurocognitive impairment, pediatric sickle cell, sub-Saharan Africa

## Abstract

**Introduction:**

The neurocognitive functions in Ugandan children aged 1–12 years with sickle cell anemia (SCA) were compared to their non-SCA siblings to identify risk factors for disease-associated impairment.

**Methods:**

This cross-sectional study of the neurocognitive functions in children with SCA (*N* = 242) and non-SCA siblings (*N* = 127) used age- and linguistically appropriate standardized tests of cognition, executive function, and attention for children ages 1–4 and 5–12. Test scores were converted to locally derived age-normalized *z*-scores. The SCA group underwent a standardized stroke examination for prior stroke and transcranial Doppler ultrasound to determine stroke risk by arterial flow velocity.

**Results:**

The SCA group was younger than their siblings (mean ages 5.46 ± 3.0 vs. 7.11 ± 3.51 years, respectively; *p* < 0.001), with a lower hemoglobin concentration (7.32 ± 1.02 vs. 12.06 ± 1.42, *p* < 0.001). The overall cognitive SCA *z*-scores were lower, −0.73 ± 0.98, vs. siblings, −0.25 ± 1.12 (*p* < 0.001), with comparable findings for executive function of −1.09 ± 0.94 vs. −0.84 ± 1.26 (*p* = 0.045), respectively. The attention *z*-scores for ages 5–12 for the SCA group and control group were similar: −0.37 ± 1.4 vs. −0.11 ± 0.17 (*p* = 0.09). The overall differences in SCA status were largely driven by the older age group, as the *z*-scores in the younger subsample did not differ from controls. Analyses revealed the strongest predictors of poor neurocognitive outcomes among the SCA sample to be the disease, age, and prior stroke (each *p* < 0.001). The impacts of anemia and SCA were indistinguishable.

**Discussion:**

Neurocognitive testing in children with SCA compared to non-SCA siblings revealed poorer SCA-associated functioning in children older than age 4. The results indicate the need for trials assessing the impact of disease modification on children with SCA.

## Introduction

Sickle cell anemia (SCA) is a serious inherited blood condition affecting 0.5%−2% of births in Uganda and other high-prevalence countries in sub-Saharan Africa (Ndeezi et al., 2016; Ware et al., 2017; Ambrose et al., 2018; Uyoga et al., 2019; Nnodu et al., 2020). A high disease burden, compounded by health and health system challenges in low-income countries, exposes many affected children to early disease complications, including cerebrovascular injury (Makani et al., 2011; Bello-Manga et al., 2016; Uyoga et al., 2019; Nnodu et al., 2021; Ranque et al., 2022). SCA-associated cerebrovascular injury commonly results in overt and/or clinically “silent” infarcts, often in children younger than 10 years of age (Bernaudin et al., 2011; Brousse et al., 2015; DeBaun and Kirkham, 2016; Munube et al., 2016; Green et al., 2019). Infarcts can lead to impaired neurocognitive functions (Kawadler et al., 2016; Prussien et al., 2019a; Knight et al., 2021; Lee et al., 2022). In high-income countries where successful stroke prevention strategies are routinely practiced, the continued occurrence of silent infarcts remains a neurocognitive risk (Bernaudin et al., 2011; Brousse et al., 2015; DeBaun and Kirkham, 2016; Kawadler et al., 2016; Kwiatkowski et al., 2019; Estcourt et al., 2020; Longoria et al., 2022). Worldwide, children with SCA with or without imaging abnormalities have a heightened risk of intellectual deficits (Prussien et al., 2019b; Idro et al., 2022).

Severe anemia is a risk factor for SCA-associated cerebral infarcts and impaired neurocognition due to abnormal blood flow and reduced cerebral oxygen delivery (DeBaun et al., 2012; Quinn and Dowling, 2012; King et al., 2014; Ford et al., 2018; Ogunsile et al., 2018; Estcourt et al., 2020; Jacob et al., 2022). The risk of cognitive impairment from SCA in sub-Saharan Africa may be compounded by low parental education, a proxy for poverty, malnutrition, and endemic infections (Dhabangi et al., 2016; Oluwole et al., 2016; Macharia et al., 2018; Prussien et al., 2019a, 2020; Bello-Manga et al., 2020). Moreover, stroke reduction strategies are not generally available in the region (Noubiap et al., 2017; Marks et al., 2018; Green et al., 2019). Cerebrovascular injury among the many African children with SCA raises questions about the prevalence and types of neurocognitive risk in this population (Marks et al., 2018). To date, few pediatric studies of SCA in sub-Saharan Africa have assessed the associated neurocognitive effects compared to unaffected children (Ruffieux et al., 2013; Oluwole et al., 2016; Prussien et al., 2019a; Jacob et al., 2022). Only one of these studies compared results to sibling controls, a strategy that can better control for environmental and socioeconomic effects (Jacob et al., 2022).

We assessed the frequency of neurological and neurocognitive impairment in a cross-sectional study of Ugandan children with SCA ages 1–12 years, “Burden and Risk of Neurological and Cognitive Impairment in Pediatric Sickle Cell Anemia in Uganda (BRAIN SAFE)” (Green et al., 2019). The overall frequency of neurocognitive dysfunction was 11.2%, with older (ages 5–12) at a 3-fold higher risk of impairment compared to younger participants (ages 1–4). In this secondary analysis, we report detailed findings of the neurocognitive evaluation of participants compared to their non-SCA siblings to identify contributions from demographic and clinical factors beyond age. We hypothesized that, compared to non-SCA siblings, children with SCA had lower neurocognitive functioning and that age, malnutrition, adverse neurological outcomes of prior stroke, and elevated transcranial Doppler (TCD) ultrasound velocity were risk factors. In contrast to other sub-Saharan Africa studies of children with SCA, we assessed the detailed neurocognitive performance for cognition, executive function, and attention in a large sample of Ugandan children compared to non-SCA siblings, as well as the effects of key demographic and neurological risk factors.

## Materials and methods

### Study design and setting

A random cross-sectional sample of 265 children with SCA ages 1–12 years attending the Mulago Hospital Sickle Cell Clinic in Kampala, Uganda, and a sample of their non-SCA siblings were enrolled in BRAIN SAFE 1 (2016–2018) (Green et al., 2019). The sample size was determined from previously reported frequencies and impacts of cerebral infarction on neurological and neurocognitive functions (Kawadler et al., 2016; Prussien et al., 2019a; Knight et al., 2021; Lee et al., 2022). Routine SCA pediatric care did not include disease-modifying therapy at that time. The study was approved by the Makerere University School of Medicine Research and Ethics Committee, the Uganda National Council for Science and Technology, and the Columbia University Institutional Review Board.

### Participants

As previously reported, inclusion criteria were (a) SCA confirmed by hemoglobin electrophoresis (HbSS or HbS-B^0^ thalassemia) and (b) having attended the Mulago SCA clinic (Green et al., 2019). To focus on SCA-related neurological complications, we excluded those with a history of neurological abnormalities before 4 months of age (Bainbridge et al., 1985). Caregiver written informed consent was obtained, with assent from participants aged 8 years or older. Non-SCA participants (*N* = 127) were also enrolled, with inclusion criteria of (a) aged 1–12 years and (b) hemoglobin electrophoresis demonstrating a lack of SCA (i.e., HbAA or HbAS). Among these controls, 119 (93.7%) were siblings; the rest were other close relatives or neighbors. Hence, we refer to them as “siblings.”

### Physical and neurological assessments and caregiver education

The World Health Organization (WHO) standards were used for the anthropometric assessments for malnutrition of the SCA and sibling participants to detect malnutrition, defined as low weight-for-height (“wasting”), as previously reported (Duggan, 2010; Green et al., 2019). The assessments of SCA participants at enrollment were a medical history and physical examination, an examination for prior stroke using the National Institutes of Health (NIH) Pediatric Stroke Scale, and stroke risk stratification by intracranial arterial flow velocity identified by TCD as elevated to ≥170 cm/s (“conditional” or “abnormal”) (Green et al., 2019). Caregiver educational attainment was scored as previously performed, reported as none, primary school, secondary school, more than secondary education, or unknown (Bangirana et al., 2009).

### Neurocognitive assessment

Overall neurocognitive functioning, including behavioral measures, attention, and executive function, was assessed using age-appropriate tests by experienced testers in both SCA and non-SCA siblings. All assessment tools had previously been translated into the predominant local language, validated, and used to establish age-specific community norms for healthy children in Kampala (Green et al., 2019). These tools have also been used to assess cognitive outcomes after severe malaria and pediatric HIV within the same community (Familiar et al., 2015; Hickson et al., 2019).

For children aged 1–4 years, the Mullen Scales of Early Learning (Mullen) and the Behavioral Rating Inventory for Executive Function–Preschool version (BRIEF-P) assessed cognitive functioning and executive function, respectively (Gioia et al., 2002; Boivin et al., 2013). The Mullen subtests assess gross and fine motor, visual reception, receptive language, and expression language. A summation of fine motor, visual reception, receptive language, and expressive language scores constitute the Early Learning Composite for measuring overall neurocognitive ability, the primary outcome for the Mullen. The BRIEF-P is a caregiver assessment of the child's executive functioning using 63 items for which the caregiver endorses child behaviors exhibited over the prior 6 months. The summation of these items gives a Global Executive Composite for measuring executive function, the primary outcome of the BRIEF-P. The subtests were for self-control, flexibility, and metacognition.

Children aged 5–12 years were tested using the Kaufman Assessment Battery for Children, Second Edition (KABC-II) (Tumwine et al., 2018), the BRIEF school-age version (Gioia et al., 2002), and the Test of Variables of Attention (TOVA) (Bangirana et al., 2009) to assess overcall neurocognitive functioning, executive function, and attention, respectively. The KABC-II subscales assessed working memory, visual-spatial ability, learning ability, and reasoning. A summation of these four scales generates a composite value, the Global Mental Processing Index. The BRIEF for school-age participants uses caregiver responses on 86 items. Here, the composite score, computed from the subtests of behavioral regulation and metacognition, generated the General Executive Composite. The TOVA, a computerized test for which children are instructed to press a switch whenever a specific target appears on the screen, assesses attention and inhibitory control. The composite score, D-prime, is calculated from subtest scores for omission errors, commission errors, response time, and attention-deficit/hyperactivity disorder.

### Statistical analyses

SCA and non-SCA participants were grouped into two age ranges according to the tests used. The raw scores for all neurocognitive assessments were converted to age-normalized *z*-scores using the established standards for unaffected healthy children, as previously described (Green et al., 2019). Within each age range, the *z*-scores were analyzed and compared, by group, using means and standard deviations. Negative values correspond to scores below the age-normalized *z*-scores. In contrast, negative *z*-scores for the BRIEF and the BRIEF-P indicate better function. Hence, the positive signs for those two BRIEF tests were flipped to negative for a consistent directionality in reporting the results (Hickson et al., 2019). Data were analyzed using means and standard deviation. For categorical data, an analysis of variation and a Pearson chi-square test were used for analyses. Continuous data were analyzed using the Pearson correlation. The factors associated with impaired neurocognition were assessed using linear regression. No missing participant data were imputed.

## Results

### SCA and non-SCA siblings demographic and clinical characteristics

Neurocognitive assessment was performed on 242 of 265 (91.3%) SCA participants and all 127 non-SCA siblings. The parents of the 23 SCA participants without neurocognitive assessments were unable to schedule testing (Green et al., 2019). The mean age of SCA participants tested was 5.46 ± 2.98 years vs. 7.11 ± 3.51 in the non-SCA siblings (*p* < 0.001; Table 1). The mean hemoglobin concentration was also highly different by group: 7.32 ± 1.02 vs. 12.06 ± 1.42 in the SCA vs. control group, respectively (*p* < 0.001). Close to half (48.4%) of the SCA sample was female compared to 43.9% of the non-SCA siblings (*p* = 0.33). Malnutrition, defined as weight-for-age at −2 *z*-scores or below using WHO global norms by age and sex, was found in 37 (15.3%) children with SCA and 11 (8.7%) controls (*p* = 0.20). Caregiver education differed between the two study groups (*p* = 0.018), with a higher proportion of caregivers in the control group having little or no education.

**Table 1:** Demographic and neurologic characteristics stratified by SCA status and age group, 1–4 or 5–12 years of age.

	**Total sample**			**SCA by age group**			**Non-SCA sibs by age group**		
	**Total SCA sample (*****N*** = **242)**	**Non-SCA Siblings (*****N*** = **127)**	***p*** **-value**	**Ages 1–4 years (*****n*** = **100)**	**Ages 5–12 years (*****n*** = **142)**	***p*** **-value**	**Ages 1–4 years (*****n*** = **40)**	**Ages 5–12 years (*****n*** = **87)**	***p*** **-value**
Age years, mean ±*SD*	5.46 ±2.98	7.11 ± 3.51	**< 0.001**	2.65 ± 0.85	7.40 ± 2.05	**< 0.001**	2.53 ± 1.12	8.34 ± 2.52	**< 0.001**
Hemoglobin (g/dl), mean (*SD*)	7.32 ± 1.02	12.06 ± 1.42	**< 0.001**	7.35 ± 1.11	7.29 ± 0.96	0.71	10.94 ± 1.55	12.6 ± 1.06	**0.001**
Female, *N* (%)	118 (48.4%)	55 (43.9%)	0.33	41 (41%)	75 (52.8%)	**0.02**	16 (40%)	39 (44%)	0.39
Malnutrition,^a^ *N* (%)	37 (15.3%)	11 (8.7%)	0.20	10 (10.3%)	27 (20.3%)	**0.04**	3 (7.5%)	8 (9.2%)	0.74
Caregiver education, *N* (%)			**0.018**			0.12			0.14
None/primary school	178 (73.6%)	109 (85.8%)		74 (74%)	104 (73.2%)		38 (95%)	71 (81.6%)	
Secondary/ tertiary school	56 (23.1%)	18 (14.2%)		23 (23%)	33 (23.2%)		2 (5%)	16 (18.4%)	
Unknown	8 (3.3%)	0		3 (3.0%)	5 (3.5%)		0	0	
Stroke by exam, *N* (%)	15 (6.2%)	–	n/a	3 (3.0%)	12 (8.5%)	**0.05**	–	–	–
Elevated TCD,^b^ *N* (%)	37 (15.5%)	–	n/a	18 (18.0%)	19 (13.4%)	0.08	–	–	–

Data are expressed as *N* (%) unless otherwise stated. Bold font represents statistically significant differences (*p* ≤ 0.05). SCA, sickle cell anemia; TCD, transcranial Doppler; n/a, not applicable. ^a^Defined by World Health Organization standards for wasting, defined as weight-for-age of −2 *z*-score or lower (Duggan, 2010). ^b^Excluding 11 children with SCA: 1 who was uncooperative, 1 aged < 2 years for whom TCD standards have not been established, and 9 with inadequate temporal bone windows for obtaining TCD velocity.

As the neurocognitive testing platforms used differed by the two age groups, ages 1–4 and 5–12, key variables were compared within the group of children with SCA and the controls (Table 1). For the SCA sample, the older group had higher proportions of females and malnutrition compared to the younger group. Among neurological outcomes in the SCA sample, a higher proportion of the older group had a prior stroke and a marginally lower proportion had an elevated TCD velocity. Among the controls, the older group differed only in having a significantly higher mean hemoglobin concentration.

Supplementary Table S1 compares each age group by SCA status. As in the overall sample, higher hemoglobin levels were found in the control group for each age group. No other significant differences were found between the younger groups. In contrast, the older SCA group was younger than the control group, had a larger proportion of malnutrition, and higher caregiver education.

### Overall neurocognition in children with SCA vs. non-SCA siblings

**Figure 1:**
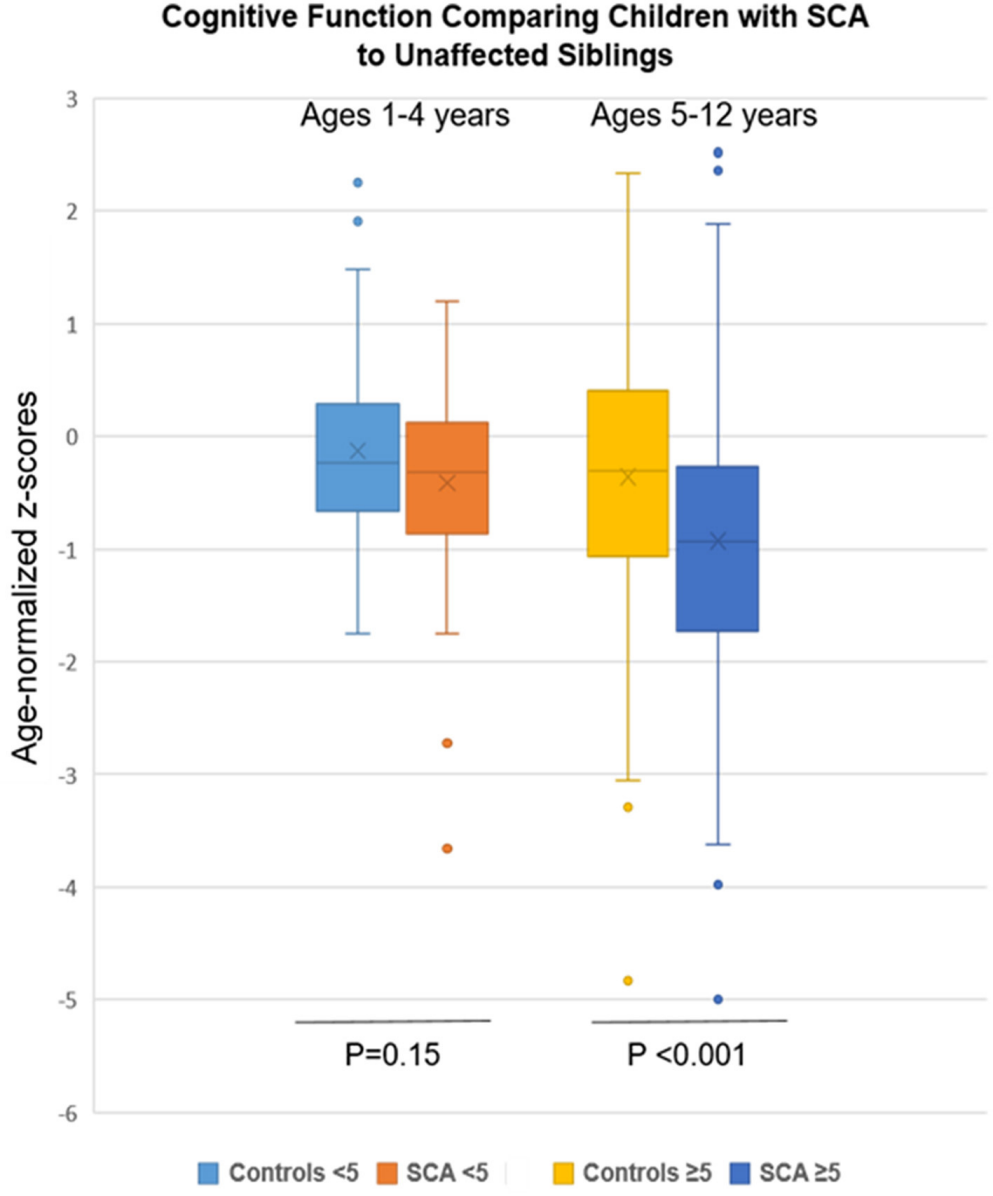
Cognitive findings by SCA status and age group (1–4 and 5–12 years) compared to unaffected siblings. Age-normalized *z*-scores for mean cognitive testing were lower in the SCA group but only in the older age group (*p* < 0.001).

The mean scores for the groups with SCA and non-SCA siblings were normally distributed for all three neurocognitive domains tested. The overall sample with SCA performed significantly worse for cognition in the standardized age-appropriate tests than the non-SCA controls, −0.73 ± 0.98 vs. −0.25 ± 1.12 (*p* < 0.001; Table 2, Figure 1). Similarly, the SCA sample scored lower for executive function than their unaffected siblings: −1.09 ± 0.94 vs. −0.84 ± 1.26 (*p* = 0.045), respectively.

**Table 2:** Composite neurocognitive test results, by z-scores, for children aged 1–4 and 5–12 years, assessed by tests for cognition, executive function and attention (the last test was used only with the older group).

**Overall differences by sickle cell status, aged 1–12 years**			
	SCA participants (*N* = 242)	Non-SCA siblings (*N* = 127)	
	Mean ±*SD* (Range)	Mean ±*SD* (Range)	*p*-value
Overall cognition	−0.73 ± 0.98 (-5.0-2.51)	−0.25 ± 1.12 (-3.84-3.42)	**< 0.001**
Overall executive function	−1.09 ± 0.94 (-2.56-3.25)	−0.84 ± 1.26 (-2.87-3.35)	**0.045**
**Differences by sickle cell status, aged 1–4 Years**			
	SCA participants (*N* = 100)	Non-SCA siblings (*N* = 40)	
	Mean (*SD*) (Range)	Mean (*SD*) (Range)	
Mullen scales of early learning^a^	−0.41 ± 0.73 (-3.66, 1.20)	−0.21 ± 0.84 (-1.65-3.42)	0.15
BRIEF-P: global executive function^a, b^	−1.47 ± 0.89 (-1.01-3.25)	−1.61 ± 1.22 (-2.87-3.35)	0.44
**Differences by sickle cell status, aged 5–12 years**			
	SCA participants (*N* = 142)	Non-SCA siblings (*N* = 87)	
	Mean (*SD*) (Range)	Mean (*SD*) (Range)	
KABC-II mental processing index^a^	−0.93 ± 1.25 (−5.00–2.52)	−0.36 ± 1.24 (−3.84–3.16)	**< 0.001**
BRIEF: global executive function^a, b^	−0.69 ± 0.83 (-2.56–2.05)	−0.32 ± 0.99 (−2.81–1.86)	**0.009**
TOVA: D' Prime^a, c^	−0.37 ± 1.4 (-3.31–1.80)	−0.11 ± 0.17 (−3.10–5.00)	0.09

Standardized age-normalized z-scores were scored based on local unaffected controls.^1^ (Ref. Bangirana et al., 2009; Green et al., 2019).

^a^The 2 to 5 component sub-tests for each neurocognitive test are listed in the Methods.

^b^BRIEF-P and BRIEF scores were flipped from positive to negative for the for consistent data directionality.

^c^This test is not valid in children below age 5.

Bold font represents statistically significant differences (*p* ≤ 0.05).

Among those in the younger age group, ages 1–4 years, who were tested, no differences according to SCA status were found in cognition or executive function. Cognitive function, tested using the Mullen, was −0.41 ± 0.73 for SCA and −0.21 ± 0.84 (*p* = 0.15) for non-SCA samples. Similarly, executive function, tested using the BRIEF-P in these younger participants comparing SCA vs. non-SCA was −1.47 ± 0.89 vs. −1.61 ± 1.22 (*p* = 0.44), respectively. Hence, neurocognitive function was retained in the younger subsample of children with SCA through age 4.

In contrast, lower *z*-scores were found according to SCA status for the older children for neurocognition, tested using the KABC-II, −0.93 ± 1.25 vs. −0.36 ± 1.24 (*p* < 0.001), and for executive function, tested using the BRIEF, −0.69 ± 0.83 vs. −0.32 ± 0.99 (*p* = 0.009). However, testing for attention, using TOVA, which was possible only among the older age group, demonstrated a similar performance between the two groups: −0.37 ± 1.4 vs. −0.11 ± 0.17 (*p* = 0.09) for the SCA and control groups, respectively. Two of the three areas of assessment, cognition and executive function, were lower in the older subsample of children with SCA compared to controls.

To remove the potential of excess influence on cognition from prior stroke within the SCA sample, we reanalyzed the mean *z*-scores after removing 15 affected SCA participants (mean age 6.0 ± 2.59 years). As expected, the mean *z*-score for the SCA subsample with prior stroke vs. no stroke was much lower −2.18 ± 1.53 than the overall scores (*p* < 0.001). However, removing this small subset affected by stroke from the SCA sample had no significant effects on the mean SCA *z*-scores for cognition or executive function compared to controls.

### Factors associated with impaired cognition in SCA children vs. non-SCA siblings

The overall neurocognitive test results for each of the domains for SCA were compared to controls for each variable collected. We first asked whether hemoglobin concentration for the SCA group compared to controls had effects that were separable from SCA in neurocognitive outcomes. Using linear regression with both variables in the model—SCA status and hemoglobin concentration—the difference was *p* = 0.004, with the main effect largely driven by SCA. No effects from sex, malnutrition, or elevated TCD velocity were found in any of the three neurocognitive domains tested (Table 3).

**Table 3:** Neurocognitive outcomes compared between the groups with SCA and controls.

**Cognition**	***F*-Value**	***p*-Value**
Unadjusted	16.1	**< 0.001**
Model 1: Adjusted for age	15.0	**< 0.001**
Model 2: Adjusted for age, hemoglobin, caregiver education^a^	8.5	**< 0.001**
Model 3: Adjusted for age, hemoglobin, caregiver education, prior stroke^a^	12.6	**< 0.001**
**Executive function**		
Unadjusted	10.8	**0.001**
Model 1: Adjusted for age	5.4	**0.005**
Model 2: Adjusted for age, hemoglobin, caregiver education^a^	2.6	**0.034**
Model 3: Adjusted for age, hemoglobin, caregiver education, prior stroke^a^	2.3	**0.04**
**Attention**		
Unadjusted	2.9	0.09
Model 1: Adjusted for age	3.6	**0.03**
Model 2: Adjusted for age, hemoglobin, caregiver education^a^	2.0	0.10
Model 3: Adjusted for age, hemoglobin, caregiver education, prior stroke^a^	2.6	**0.028**

By linear regression, adjusting for age, hemoglobin, caregiver education and prior stroke changed affect sizes but not outcomes of cognition or executive function, thereby identifying SCA as the primary source of group differences. ^*^In contrast, attention was largely influenced by SCA and age differences between the two samples.

^*^These variables had no effect on outcomes: sex, malnutrition, elevated TCD velocity. Caregiver education had a modestly significant impact on executive function.

^a^ Hemoglobin concentration did not impact the variance beyond that from SCA.

Bold font represents statistically significant differences (*p* ≤ 0.05).

We then examined overall test outcomes for factors contributing to the outcomes (Table 3). For overall cognition or executive function, age-normalized *z*-scores declined with age. Adjusting for other variables of hemoglobin concentration, caregiver education, and prior stroke demonstrated their impact on effect sizes. Despite those changes, the outcomes were unchanged. These findings demonstrated that the poorer function of the SCA group was attributed to the impact of the disease.

By the TOVA test, for attention in the older participants in both SCA and controls, age also negatively impacted *z*-scores. The influence of age for the SCA group was assessed as *r*^2^ = −0.58 and −0.72 for the controls (both *p* < 0.001). Unexpectedly, neither prior stroke nor elevated TCD was associated with reduced attention.

Finally, we examined the SCA sample for impact from prior stroke or elevated TCD velocity on each of the three outcomes. Prior stroke strongly affected cognition (*p* < 0.001) but had no significant effects on executive function or attention (Table 4). Elevated TCD velocity had a borderline impact on cognition, but it, like prior stroke, had no impact on executive function or attention.

**Table 4:** Examining the role of prior stroke or elevated TCD velocity on neurocognitive outcomes within the SCA sample.

**Prior stroke**	**F-value**	***p*-value**
Cognition	25.1	**< 0.001**
Executive function	0.06	0.43
Attention	0.14	0.71
**Elevated TCD Velocity**		
Cognition	3.4	0.067
Executive function	0.23	0.63
Attention	0.01	0.91

By linear regression of age-normalized z-scores, adjusting for prior stroke demonstrated a strong relationship with cognition but not the other outcomes. In contrast, adjusting for elevated TCD velocity demonstrated that no outcome was affected^*^.

^*^1 participant had both prior stroke and elevated TCD velocity.

Bold font represents statistically significant differences (*p* ≤ 0.05).

## Discussion

Children with SCA in sub-Saharan Africa are at risk for disease-associated cerebrovascular injury as well as environmental challenges (Oron et al., 2020; Nnodu et al., 2021). In a large clinic-based sample of Ugandan children with SCA in Kampala compared to their non-SCA siblings, our cross-sectional neurocognitive assessment revealed these main findings: (1) The mean test *z*-scores for cognition and executive function were substantially lower in the SCA sample, even after accounting for age, hemoglobin concentration, caregiver education, and prior stroke. No discernable effects were seen regarding sex, malnutrition, or elevated TCD velocity. These findings confirm SCA as the main cause of impaired neurocognition in this study. Lower scores in cognition testing of approximately 0.5 *z*-scores in the children with SCA correspond to approximately 8 IQ points below that of their siblings. (2) The differences by hemoglobinopathy diagnosis were driven by the older subsample with SCA, ages 5–12 (Gur et al., 2021). In contrast, children with SCA aged 1–4 were not different in cognition or executive function from unaffected siblings in that age group. (3) Age and clinically evident prior stroke, but not elevated TCD arterial velocity, were the largest drivers of impaired cognition. Nonetheless, excluding a modest number of SCA participants with prior stroke from the analyses did not significantly affect the results of the remaining sample. (4) Among those aged 5–12, attention was not significantly different from the controls unless accounting for differences in age and/or prior stroke. (5) The non-SCA siblings in our study scored below healthy Kampala-based age-normalized controls. What social, economic, and/or educational opportunities affected neurocognitive performance in the siblings was not assessed.

Similar findings of impaired neurocognitive function and attention of older SCA children compared to non-SCA siblings were observed in a prior Tanzanian report of a smaller sample (Jacob et al., 2022). Similar to prior reports of children with SCA in Africa, the United States, and elsewhere, our study's participants with SCA had lower executive functioning (Ruffieux et al., 2013; Prussien et al., 2019a; Jacob et al., 2022). This consistent finding was seen despite our use of a test by parental report, which may under-report functional deficits, rather than direct testing used by other studies (Prussien et al., 2019a; Trpchevska et al., 2022).

Unlike our findings here, the Tanzanian study did not observe a decline in performance in older vs. younger SCA participants (Jacob et al., 2022). As that study tested children aged 6 years and older, taken together, these findings support our findings of the sparing and/or resilience of the younger age group with SCA. Consistent with this observation, the cumulative effects of SCA cerebrovascular injury over time are considered to be primarily responsible for the association between age and neurocognitive impairment in children with SCA (Wang et al., 2001; Schatz and McClellan, 2006). Collectively, these observations suggest that low caregiver education, alone and/or as a surrogate for low socioeconomic status (SES), and/or educational disadvantages may contribute to—but are not the main drivers of—the age effects seen in children with SCA in sub-Saharan Africa (Bangirana et al., 2009). Similar findings were reported in a U.S. study, in which SCA and social factors both influenced neurocognition (King et al., 2014). The effect of age on the neurocognitive outcome in our study could also be a consequence of “growing into deficit,” whereby effects of brain injury become apparent as the child grows older (Zhuo et al., 2022).

A relatively high proportion of neurocognitive impairment in the older group of non-SCA siblings may be at least attributable to social issues, as all neurocognitively impaired siblings had caregivers with low education. Our group has previously reported this association among healthy children in Kampala (Bangirana et al., 2009).

This study's limitations include potential biases from SCA-associated survival and the cross-sectional study design. These issues may have affected the relationships seen with age. Nonetheless, our data reflect results from a substantial number of children receiving SCA care at a large urban center. Executive function was based on parental report rather than direct child assessment; hence, it could have been biased. More direct measures of executive function may provide clearer insights. A test for attention comparable to the TOVA for children ages 1–4 years was not available. Executive function was not tested directly, as the BRIEF and BRIEF-P use parental reports. At that time, we had no other option for which local translated platforms and age-normalized standards were available. We have previously used these tests to assess executive function in local studies on childhood malaria and HIV infection (Familiar et al., 2015; Hickson et al., 2019). Additional limitations include potential differential influences from illness-associated school absences adversely impacting test results and no assessment of attention in the younger age group (Olatunya et al., 2018). The contributions from hemoglobin concentration could not be discerned from SCA as they were tightly linked. Unlike the random SCA clinic-based selection, sibling participation may have been biased, for example, from possible parental concerns. The potential for downward socioeconomic pressure associated with having a child affected by SCA may have contributed to subnormal scores among the siblings (Amarachukwu et al., 2022). Low caretaker education level, a marker of poverty, may have adversely affected neurocognitive scores, although the sibling assessment would have modulated those effects (King et al., 2014; Bello-Manga et al., 2020; Jacob et al., 2022). No adjustments were made for multiple comparisons.

In conclusion, comparing a sample of Ugandan children with SCA to their non-SCA siblings aged 1–12 years, we demonstrated that children with SCA had worse cognitive impairment and executive function than the unaffected siblings and that these differences were attributable to the older age group, aged 5–12. The younger children with SCA were not different from their non-SCA siblings. Age 5–12 and prior stroke were most strongly associated with neurocognitive impairment, with some contribution from caregiver educational attainment. Low neurocognitive *z*-scores by age among non-SCA siblings suggest environmental influences, for example, SES and education, among all participants, with potential parental selection bias for the siblings tested. Given the increased risk of impairment with age, interventions in early childhood may more likely provide benefits. Disease-modifying therapies, for example, hydroxyurea, should be tested for stabilizing or improving neurocognitive functions in young sub-Saharan children through amelioration of modifiable risk factors with SCA, including anemia (Tshilolo et al., 2019; Opoka et al., 2020).

## Data availability statement

The data supporting the conclusions of this article will be made available by the authors, in accordance with appropriate protection of the privacy of participants.

## Ethics statement

The studies involving humans were approved by the Makerere University School of Medicine Research and Ethics Committee and the Columbia University Institutional Review Board. The studies were conducted in accordance with the local legislation and institutional requirements. Written informed consent for participation in this study was provided by the participants' legal guardians/next of kin. Written informed consent was obtained from the minor(s)' legal guardian/next of kin for the publication of any potentially identifiable images or data included in this article.

## Author contributions

PB: Conceptualization, Formal analysis, Investigation, Methodology, Resources, Supervision, Writing – review & editing. AKB: Formal analysis, Writing – original draft, Writing – review & editing. AB: Data curation, Formal analysis, Methodology, Supervision, Writing – review & editing. RO: Conceptualization, Investigation, Methodology, Project administration, Resources, Supervision, Writing – review & editing. DM: Conceptualization, Supervision, Writing – review & editing. EM: Conceptualization, Methodology, Resources, Supervision, Writing – review & editing. PK: Resources, Supervision, Writing – review & editing. GM: Data curation, Investigation, Project administration, Supervision, Writing – review & editing. MM: Data curation, Investigation, Project administration, Supervision, Writing – review & editing. GR: Investigation, Writing – review & editing. NG: Conceptualization, Funding acquisition, Investigation, Supervision, Writing – original draft, Writing – review & editing. RI: Conceptualization, Investigation, Project administration, Resources, Supervision, Writing – original draft, Writing – review & editing.
