# Supplementary material for: Neurocognitive impairment in Ugandan children with sickle cell anemia compared to sibling controls: a cross-sectional study
Source: Front Stroke. 2024 Apr 15;3:1372949. doi: 10.3389/fstro.2024.1372949 (PMC11188974; doi:10.3389/fstro.2024.1372949)
Supplement: Supplementary file 1 [file Table_1.docx]

**Supplemental Table. Demographic characteristics stratified by age group, 1-4 or 5-12 years of age, comparing participants with SCA to controls.** Data are expressed as N (%) unless otherwise stated.

|  | **SCA** | **Non-SCA Sibs** | **p-value** |
| --- | --- | --- | --- |
| **Ages 1- 4 years** | N=100 | N=40 |  |
| Age years, mean ±SD | 2.65±0.85 | 2.53 ±1.12 | .43 |
| Hemoglobin (g/dl), mean (SD) | 7.35 ±1.11 | 10.94 ±1.55 | **<.001** |
| Female, N (%) | 41 (41%) | 16 (40%) | .73 |
| Malnutrition,^1^ N (%) | 10 (10.3%) | 3 (7.5%) | .08 |
| Caregiver education, N (%) |  |  | .38 |
| None/Primary School | 74 (74%) | 38 (95%) |  |
| Secondary/ Tertiary School | 23 (23%) | 2 (5%) |  |
| Unknown | 3 (3.0%) | 0 |  |
| **Ages 5-12 years** | N=142 | N=87 |  |
| Age years, mean ±SD | 7.40 ±2.05 | 8.34 ±2.52 | **.04** |
| Hemoglobin (g/dl), mean (SD) | 7.29 ±.96 | 12.6±1.06 | **<.001** |
| Female, N (%) | 75 (52.8%) | 39 (44%) | .79 |
| Malnutrition,^1^ N (%) | 27 (20.3%) | 8 (9.2%) | **<.001** |
| Caregiver education, N (%) |  |  | **.03** |
| None/Primary School | 104 (73.2%) | 71 (81.6%) |  |
| Secondary/  Tertiary School | 33 (23.2%) | 16 (18.4%) |  |
| Unknown | 5 (3.5%) | 0 |  |

^1^Defined by WHO standards for wasting, defined as weight-for-age of -2 z-score 

 or lower (ref. 39)

 Bold font represents significant differences (≤0.05).
